# Multiple Openings and Competitiveness of Forward Markets: Experimental Evidence

**DOI:** 10.1371/journal.pone.0158098

**Published:** 2016-07-21

**Authors:** José Luis Ferreira, Praveen Kujal, Stephen Rassenti

**Affiliations:** 1 Department of Economics, Universidad Carlos III de Madrid, Madrid, Spain; 2 Department of Economics, Middlesex University, London, United Kingdom; 3 Department of Economics, Chapman University, California, United States of America; University of the Basque Country, SPAIN

## Abstract

We test the competition enhancing effect of selling forward in experimental Cournot duopoly and quadropoly with multiple forward markets. We find that two forward periods yields competitive outcomes and that the results are very close to the predicted theoretical results for quantity setting duopolies and quadropolies. Our experiments lend strong support to the hypothesis that forward markets are competition enhancing. We then test a new market that allows for endogenously determined indefinitely many forward periods that only close when sellers coordinate on selling a zero amount in a forward market. We find that the outcomes under an endogenous close rule are also very competitive. These results hold for both duopolies and quadropolies.

## 1. Introduction

Do forward markets enhance competition in oligopolies? Our results, both for two forward markets [1] and endogenous close, supports the existing experimental [2–4] and empirical literature [5]. Theory, however, is not clear on the issue. [6] and [1] suggest pro-competitive outcomes while [7–9] suggest anticompetitive outcomes in models with price competition and product differentiation. Other papers have explored the various aspects of competition that may affect the strategic behavior of forward markets. For example, [10] and [11] study the observability of actions, [12] study capacity choice, [13] and [14] study entry, while [15] study regulatory arrangements to promote forward contracting.

In this paper we explore the impact of multiple openings of forward markets on its competitiveness. We explore two market structures, the first one where we study the effect on competitiveness of two forward markets and the second where we introduce a new market institution where the close of forward markets is endogenous and is determined by the decisions made by the market players.

Previous experiments tested the model in [6] that considers only one opening of the forward market. We extend these experiments to two forward market openings and observe support for the theory in two aspects. First, overall average output for both duopolies and quadropolies is very close to the theoretical prediction. Second, and even more remarkably, outputs are observed following the predicted proportional shares among the different forward and spot markets. Our experiments provide stronger evidence towards the competition enhancing effect of forward markets shown so far and strongly support the predictions of [1].

Our main contribution is the introduction of a new market structure that allows for endogenously determined indefinitely many periods in the forward stage. In our proposed experimental forward market we introduce an endogenous rule that closes the forward markets (and moves to the spot market) whenever all subjects stop using them (see details in Section 2.1). This structure allows for both the collusive and competitive equilibria. Operationally, it imposes strong cognitive requirements on sellers to collude on the quantity sold in forward market, i.e. zero, to directly go to the spot phase, for “collusion” to be successful. This makes it a very complicated environment for sellers to coordinate and we felt that this was a good (extreme) test for the competiveness of forward markets.

The experimental results show that indeed quantities are at competitive levels under an endogenous close rule. An endogenous close rule forward market yields competitive outcomes. The quantity is mainly sold in the first few openings of the forward market, leaving almost nothing to be sold in the rest of the forward (and spot) markets. As large quantities are sold in the first openings of the forward markets, the corresponding Cournot price in the residual demand gets lower, and converges to the competitive price.

The little experimental literature has suggested that forward markets enhance market competitiveness. [2] were the first ones to experimentally test [1] with a single forward and a spot market phase with fixed matching. They showed that, relative to the spot market, the introduction of forward markets has competition enhancing effects. As predicted by theory subjects avail of forward markets. Forward markets are not as competitive as theory predicts when there are two firms, but are not significantly different than the theory prediction for four firms. Spot markets, meanwhile, observe greater quantities compared to the theoretical prediction. Quadropolies, on the other hand sell more and a greater proportion of quantity is sold in the forward market.

The second experimental study [3] is based on a specific design of forward markets in the electric power industry. They consider both quantity and supply functions as strategic variables. In a model following Allaz and Vila, they study the effects of forward markets when 2 or 3 firms can submit quantities or supply functions and find that the introduction of forward markets has competition enhancing effects. Moreover, supply functions have efficiency enhancing effects in the presence of forward markets. The third experimental work [4] also tests a version of the Allaz and Vila model and reaches the same conclusion, *i*.*e*., both market quantity and efficiency increase with the introduction of a forward market.

The empirical research on forward markets is scarce. For the Australian power market, [5] shows that the effect is pro-competitive when firms use forward markets. One should, however, add a note of caution. Even though the use of forward markets is spreading, in many instances market regulation requires firms to participate in these markets. Most of the theoretical models described above agree that, when used, forward markets are pro-competitive. However, they differ in a very important respect. In some models, the equilibrium behavior forces firms into entering the forward market, while in others, there are equilibria in which firms avoid these markets. Hence, *sensu stricto*, Wolak’s conclusion is only about the subgames in which firms do enter in the forward market competition, and says nothing about the question of whether firms will avoid competition by not using them when deciding in a non-regulated market.

Even though the majority of research in quantity setting games uses fixed matching, we chose random matching as we considered that it is the best test of the one shot prediction of the theoretical model. When the stage game has only one equilibrium, its finite repetition has also a unique equilibrium, which is the repetition of the stage game’s equilibrium. Therefore, theoretically, conducting an experiment with random matching (with players playing only the stage game with the same opponent) or fixed matching (where the same players play a finite repetition of the stage game) should make no difference. However, in our experiments, only the exogenous close case has this property, while the endogenous close case has a multiplicity of equilibria. In addition to this, fixed matching may theoretically generate different equilibria if individuals depart from standard rationality assumptions, and empirically the finite repetition of a one-equilibrium game makes players behave differently from the stage game. An example of this is the experimental evidence of cooperation in finitely repeated prisoners’ dilemma [16].

The paper is structured as follows. First we present the results in Section 2 and. In Section 3 we present the experimental design. Section 4 analyzes the results in more detail. Section 5 concludes. We present the theoretical motivation behind the experiments in the Supporting Information (S2 File).

## 2. A Brief Look at Experimental Results

It will be useful to look at some summary statistics before we look at detailed results. Table 1 compares the theoretical and the average values in the endogenous close experiments. In order to make comparisons easier, we normalize all quantities as percentages of the competitive quantity.

**Table 1:** Summary Statistics (% of competitive quantity) Endogenous Close.

	Competitive	Cournot	Our experiments
2 firms	100	66.66	**97.64**
4 firms	100	80	**116.24**

The first thing one notices is that markets are extremely competitive both for two and four firms. This goes against the hypothesis that subjects can find a way to revert to the Cournot equilibrium by avoiding the use of the forward markets. We examine this in more detail in Section 5. Table 2 compares the theoretical and average values in the exogenous close experiments for both the 2 and 4-firm case (in the table *f* is the number of forward markets).

**Table 2:** Summary Statistics (% of competitive quantity), Exogenous Close.

	Perfect competition	Cournot output	Allaz and Vila Theory	Allaz and Vila Theory	Our Experiments
			*f =* 1	*f =* 2	*f =* 2
2 firms	100	66.66	80	85.71	**84.39**
4 firms	100	80	94.1	98.11	**101.37**

The first result that stands out in Table 2 is that the average behavior of subjects is in line with theory. While the average output observed for duopoly is near the prediction of the AV model, the quadropoly gives a competitive outcome. Although note that for *f =* 2, the theoretical prediction for the quadropoly is already very close to perfect competition.

## 3. Experimental Design

A total of 180 students were recruited from the undergraduate student populations from two major U.S. universities for a pre-specified two hour duration. None of the students had participated in prior Cournot oligopoly experiments. They were called one by one according to a randomly generated recruitment list and were asked to choose terminals as they entered. Subjects were not informed about the total number of periods in the experiments (Table 3). Including the instructions (see S1 File), all experiments ended in 2 hours or before. Each forward, or spot, period could run for a maximum of 45 seconds. If decisions were completed before the time limit then the experiment rolled over automatically to the next future, or spot, period. Subjects were paid a show-up fee of $7.

**Table 3:** # Groups (# subjects/group).

	Exogenous Stop		Endogenous Stop	
	Univ. 1	Univ. 2	Univ. 1	Univ. 2
Duopoly	1 (6)	4 (6)	1 (8)	4 (6)
		1(12)		1 (12)
Total subjects Duo	6	36	8	36
Quadropoly	--	4 (12)	--	4 (12)
Total subjects Quad	--	48	--	48

Under the title "The Economics of Decision Making" (IRB permit #: 1415H012, Chapman University), the Institutional Revue Board (IRB) at Chapman University provided ethical approval and approval of an electronic consent procedure for all experiments done at Economic Science Institute (ESI) that involve no deception, no audio or video taping, and no invasive procedures, or food or drugs. To be included in the ESI subject database, volunteers simply must provide electronic consent (name, student ID#, and acknowledgment of agreement to the terms of an electronic consent form they must read). Subjects for all generic decision-making experiments covered by IRB permit #: 1415H012 are then selected from the database to participate in particular experiments.

The market demand used in all the experiments was *p = 105 –q* with constant marginal cost for all firms at 15. Note that in an earlier experiment we had marginal costs set equal to zero for the quadropoly case. Some readers expressed concerns that this may have made the quadropoly more competitive. We have redone the quadropoly experiments with exactly the same parameters as duopoly, but the results are similar under both cost scenarios.

Subjects could observe past rival costs, prices and own and others’ output for any past period. Rival identity was unknown in all the experiments and subjects were randomly re-matched after each period. Subjects were explained the processes of random matching and of price determination in the instructions, and given specific examples. They were provided with a calculator showing two output choices, “mine” and “others”, and subsequent own profits. By resetting own and others’ output they could estimate how their profits vary as either one of the two output changes. Due to the extremely competitive nature of endogenous close forward markets subjects were paid a favorable exchange rate in the endogenous close experiments relative to the exogenous close experiments. Including show up fees of $7 subject earnings varied from $15.50-$30.86 in two hours.

In the endogenous close treatment, the forward market opened again in period *t* if there was at least one position in *t-*1 different from zero. We test an endogenous close model that captures the main features of the model in [7] (S2 File). The experimental design with an undefined number of forward openings has the drawback of having to exogenously decide the price at which the forward sales should be traded. As demand is automated, we have to make a choice in the equilibrium we want to test. To test for the competitive equilibrium requires setting a price equal to marginal cost for all forward sales. But this implies that subjects have no use for the forward markets, and the experiment simply becomes a standard Cournot experiment. Thus, the only meaningful hypotheses are the ones that depart from the competitive behavior. This would give higher profits by using the spot market alone, as in the best equilibrium in both the endogenous model tested here and the model in [7].

As a result we tested the Cournot equilibrium (*i*.*e*. the “collusive” hypothesis) and, accordingly, set the price equal to the Cournot price in the residual demand. In other words, if the market is open at forward period *t*, the program computes the price of the Cournot equilibrium after subtracting from the initial demand all forward quantities sold in forward periods 1 to *t*. This would be the price in the collusive hypothesis in that subgame, as it implies no use of forward markets and Cournot behavior in the spot market. Following the model, the no arbitrage condition requires this to be the equilibrium price as well in forward market *t*.

In the exogenous close treatment, there were two periods of forward markets previous to the spot market. To deal with the no-arbitrage condition in the exogenous close case, the forward market price in each of the forward markets periods is computed as the theoretical price that would prevail in the remaining periods if the theoretical model is solved with the residual demand. For example, if total of sales in the first period of forward markets is 20 then, in the exogenous close treatment, the program computes a forward market price for that period as the equilibrium price (as in [1]) with one period of forward markets and demand given by *p = A– 20 –q*. This way we can test the hypothesis whether subjects behave as in the Allaz and Vila model.

The main features of our experimental design are the following. First, we test the endogenous close treatment with indefinitely many periods. In this structure subjects need to coordinate on no-sales in a forward market to move over to the spot phase. The endogenous close treatment is very demanding for subjects as they need to implicitly coordinate with a rival making simultaneous choices. Notice the differences with [2], the first work to directly test Allaz and Vila’s model in the laboratory, where the design matching were fixed and the experiments ran for 30 periods.

Second, we chose random matching in each round as it makes collusive behavior due to repeated games effect difficult -see [17]- (Notice that, the random matching protocol makes the outcomes more competitive compared to the fixed matching. We thank one of the referees for making this important point). Also, our experiment is about behavior in the one-shot game. Though not perfect, this provides a better test of the one shot prediction. Although theoretically the finite repetition of the game, with only one equilibrium, cannot generate cooperation, there is experimental evidence that subjects may still cooperate for some rounds if the game is long enough [16]. This is relevant for the exogenous treatments where there is a single equilibrium. For the endogenous treatment, the one shot game and the repeated game generate different equilibrium due to the multiplicity in the one shot game. Given the small number of subjects they can be matched against the same partner with a positive probability, but since subjects do not observe rival identity, this makes collusion and other group behavior difficult. Nevertheless, prior to our data analysis we conduct a series of tests to make sure that subjects do not condition their behavior on the identity of the rival.

The use of the Cournot price in the residual demand is the only way to test the Cournot outcome, but at the same time, it gives that outcome a better chance. On the other hand, the use of random matching protocol is introduced to preserve the set of theoretical equilibria in the experiment, as opposed to using the fixed matching, that might bias the results towards more collusive outcomes. The choice of the random matching is thus neutral, but it can also be interpreted that by not choosing the fixed matching we are not giving the Cournot outcome its best shot. All in all, the experimental design is as neutral as possible, which in this test means some unavoidable bias towards the Cournot outcome when testing precisely this outcome. Since the findings are against the Cournot behavior the experiment holds its validity.

Third, we use two periods of forward markets for the exogenous close. We do this for two reasons. First, we were interested in whether market competitiveness increases with the number of forward markets. We chose the two period forward market implementation as it is theoretically more competitive than the one period implementation. This is especially true for the quadropoly case. Second, we were also interested in checking whether the way in which subjects distribute their sales among the different markets corresponds to the theory. In the case of the duopoly, according to the theory, individuals should distribute their sales evenly among the three markets (two forward and one spot), but in the case of the quadropoly, the theory predicts a more complicated pattern.

Finally, our exogenous duopoly experiments lasted 60 periods while the quadropoly experiments lasted 42 periods. We chose a longer time horizon to facilitate subject understanding of the environment as forward markets are cognitively complicated. While longer experiments favor learning, they can also be boring. This is especially true in the endogenous close experiments where all groups start at the same time due to random matching. It was common for subjects to wait for other to finish sometimes for several minutes. However, looking at choices in the last periods we find no evidence of sudden output changes or any end-game effects in both the exogenous and endogenous close experiments. For the endogenous close experiments the number of rounds varied depending upon the session. We were only able to obtain 32 rounds for the duopolies and between 24–28 rounds for quadropolies. Note that in a random matching structure the slowest player determines how fast the entire group progresses as the next round does not start till a random rematch is performed. To have an idea of the number of rounds we could accommodate in each session we conducted a pilot experiment where we paid attention to the time it took the subjects to complete each round.

### Data analysis

We need to make some clarifications regarding our data and the methodology adopted for data analysis. Given that we have random matching we can analyze our data treating each group as an independent observation (or we can treat each re-match in every subsequent period as yielding independent observations). In the present work we show the data using the latter approach (we also conducted the former with no difference in the results) as it allows us to exploit better the data we obtain. Notice that random matching in repeated games has been used in the experimental literature. See for example [18] and [19].

In a random matching protocol subjects are randomly re-matched in every subsequent period. If random matching yields independent observations for each subsequent re-pairing then, unlike in the fixed matching protocol, there is no such a thing as a market with fixed pairings that goes through all periods. Instead, we obtain individual observations for different pairings across time. This implies that under perfect random matching each match across periods is an independent observation. If this were to be the case then we can consider each individual as an observation. Note, however, that if one faces group size limitations, which we do, then subjects have a positive probability of being re-matched. This raises some reasonable concerns regarding whether the data we obtain is truly independent across time and matches. For example, in an indefinitely repeated two-player prisoners dilemma [19] shows that the cooperative norm does not appear in experiments where players are matched randomly whereas, it does emerge under fixed matching as players gain more experience.

Even if subject identity is unknown, something in the quantity choice in the previous market may be an indication of the identity of the rival. To make sure that this is not the case we test for the independence of individual choices. First, we test the hypothesis that a Player, say A, does not behave differently when playing against another player, B, compared with her play against another player C. A rejection of independence in this case implies that the behavior of subject A, when playing against subject B, can be assumed to be the different as when playing against subject C. This test is important to be able to use each individual as a data point. A preliminary view of correlation indices for the quantities chosen by different individuals in the same experiment shows no difference with correlation indices of quantities chosen by individuals in different experiments.

We performed this test on the exogenous case duopoly experiments where the possibility of re-matching is higher (where we conducted five experiments with 6 subjects each and one experiment with 12 subjects). There are 10 possible comparisons for each one of the 6 players in each one of the 3 openings of the markets in each one of the 5 experiments with 6-subjects. This gives a total of 900 tests for the 6-subject exogenous close duopoly experiments. Out of the possible 900 tests, we conducted 532 and rejected the hypothesis that players did not play differently depending on the rival in only 71 of them (7.8% of the total) at 5% significance level. Note, however, that the 71 cases may only mean that players play differently when changing the period of play, an event that may correlate with the change of rival. In quadropolies, the same set of opponents is almost never seen twice, and in the endogenous close experiments, there are fewer periods. Indeed, the tests in these other cases show even less difference in play, the problem of group effects is smaller in these cases.

Given this we run a collinearity test to see whether the choices of an individual are orthogonal to the choices of another. To check that the identity of the rival is irrelevant for the change of play (the identity of the opponent is never revealed) we conduct tests to see if the play of one player contributes to the play of a rival. For this purpose we calculate the Variance Inflation Factor (VIF) using the Collin command in Stata. VIF is the inverse of the tolerance defined as (1 –R_j_^2^) where R_j_^2^ is the coefficient of determination of an explanator *j* on other explanators. VIF values close to 1 (and below 10) indicates little multi-collinearity. We find that all VIF values for the collinearity tests are well below 2 with most of them being close to one. This confirms that in all cases the play of one player is orthogonal to the play of the other. This implies the first is irrelevant to explain the play of the second. Given the evidence presented by these tests, we treat individual plays as independent data. All tests are available upon request.

## 4. A Closer Look at the Data

### 4.1 Endogenous close: Duopoly

The design of the endogenous case needs some justification. First, it should be stressed that we do not claim to test the model in [7], but a model that shares many of its characteristics, as the model in [7] cannot be directly implemented in the laboratory.

In Ferreira’s model there is a demand corresponding to the time interval [0,1], and the market opens at time 1, where all physical transactions take place. Firms compete *a la* Cournot in a market with homogenous products. Previous to this spot market, they can sell some quantity in a forward market. The forward market opens at infinitely many times in the interval. At each of the openings firms choose how much to sell to interested buyers. After the first forward market closes, all agents know sales and prices, and then make plans for the second opening of the forward market. The model is like the Allaz and Vila model detailed in Supporting Information (S2 File), but with infinitely many periods of forward markets.

[7] shows that any price between the competitive and the Cournot prices can be obtained in a subgame perfect equilibrium. The competitive equilibrium corresponds to the limit case of the model in [1] when the number of forward periods goes to infinite. The only difference is that in [1] the quantity is divided in a precise way between each of the markets (which implies infinitesimal quantities in each opening as the number of openings increases), whereas in [7] any distribution of the quantity among the different markets can be sustained. The Cournot result is achieved when firms refrain from using the forward market and wait until the spot market. This result can be obtained in equilibrium if firms use a punishment strategy in which firms refrain from using the forward market as long as the others do the same, but sell a certain quantity if in the past period some firm used it.

The two practical reasons why [7] cannot be tested in the lab are the following. First, we cannot have infinitely many periods. Second, we face an integer problem, as many of the subgames of the original require arbitrary divisions of the money unit. We therefore construct a new model that is different from [7] in one way: instead of having infinitely many forward market openings we have a forward market that opens once, and keeps opening at time *t* as long as at time *t–* 1 some firm sold some quantity. Otherwise, the forward market does not open anymore and the game goes to the spot and last market. This endogenous close model is described in detail in Supporting Information (S2 File). Below we point out the similarities with [7]:

1. *There are multiple equilibria*, *including the competitive and the Cournot results*.
2. *In the competitive equilibrium*, *prices are zero in all forward markets*.
3. *In the Cournot equilibrium*, *firms refrain from using the forward market*.

Cournot competition with multiple openings include models of endogenous choice of the time at which to sell [20]. In this model, two firms can sell at periods 1 or 2. In the latter case they observe the other firm’s behavior at time 1. The theoretical result is that in equilibrium one of the firms chooses to sell at time 1, and becomes the Stackelberg leader, and the other chooses to sell at time 2, and becomes the follower. However, the experimental results do not agree with the theory. [21] show that subjects stick to the Cournot outcome.

While in this model firms can only sell in one of the two periods, in [1], [7] and in the endogenous close model presented in this paper, firms can choose to sell in all periods in which the market opens. In addition, in the endogenous close model, firms endogenously decide when to close the forward market to go to the spot, while in [20] they decide endogenously decide whether to compete *a la* Stackelberg or *a la* Cournot. The two types of models address very different questions and get different results. There is still a similarity in the fact that the theoretical results require solving a coordination problem, as in the endogenous close game firms need to learn to coordinate to refrain from using the forward market if they want the Cournot outcome in order to avoid more competitive ones. This failure in firms’ coordination seems to be at the heart of the discrepancies between the theoretical predictions and the experimental results in both cases.

The introduction of a finite number of forward market periods makes the firm enter a prisoners’ dilemma, if they both use the forward market they end up selling more at a lower price and make lower profits. No matter what the other firm does, each firm’s best reply is to sell in the forward market. The endogenous close rule captures the dynamic version of the prisoners’ dilemma as subjects observe rival choices (individual for duopoly and aggregate for quadropoly) and can react to them in the next forward period. Even if the game is not repeated, the forward market interaction shares some of the features that allow for cooperation if players do not make use of the forward market. In fact, the game has multiple equilibria, including both the competitive and the Cournot outcomes.

**Fig 1:**
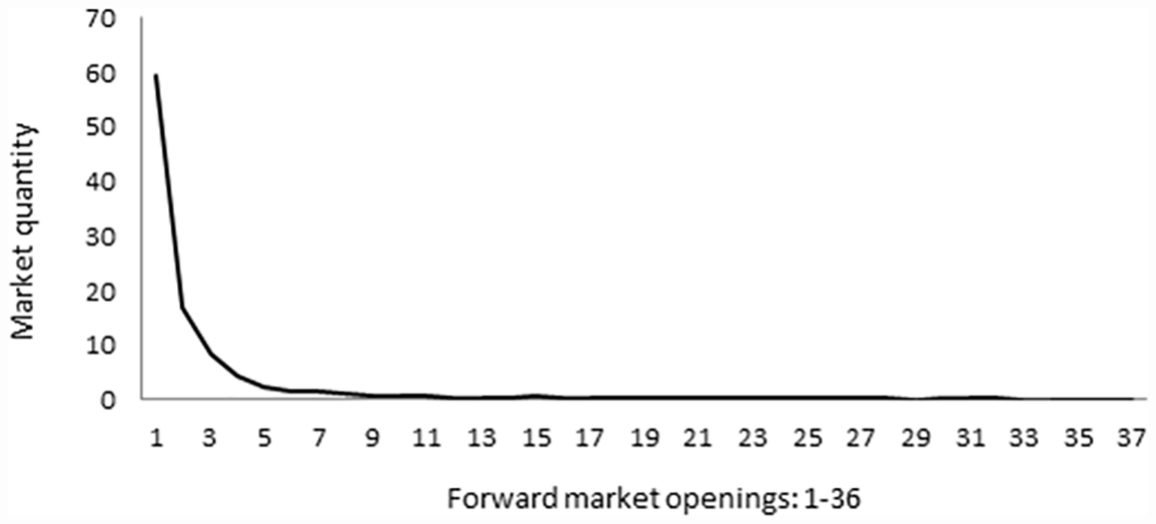
Use of forward and spot markets. Endogenous close, Duopoly.

**Fig 2:**
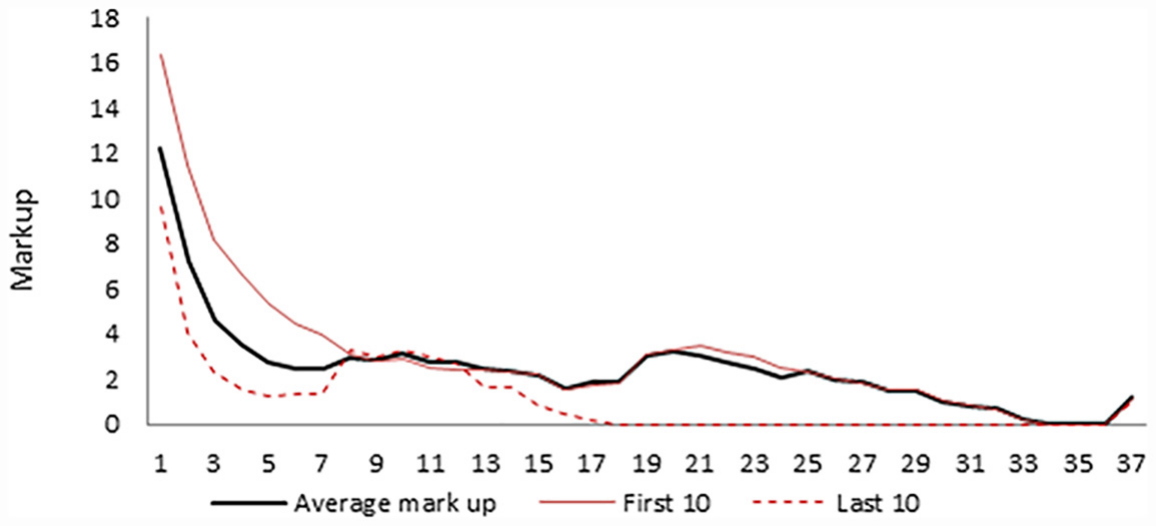
Markup per market opening. Endogenous close, Duopoly.

We designed the experiment to also test the hypothesis whether subjects are able to coordinate and stop using the forward market. This would imply that if all the sales take place in the spot market then subjects behave as a Cournot oligopoly. We observe, however, that the findings are completely at odds with this hypothesis. Both market structures, duopoly and quadropoly, behave competitively. Contrary to what has been observed in other oligopoly experiments, our duopolist subjects are very competitive, and choose quantities close to the competitive equilibrium (though significantly different). Further, sales occur almost entirely in the early forward markets with more than half of the sales in the very first opening (see Fig 1 and Table 4). Accordingly, prices converge rapidly to the competitive price. In Fig 2 we observe that the mark up (price minus marginal cost) is very small by the fourth opening of forward markets (as a reference, the Cournot markup is 30.) As the residual demand is very small in the spot market it is hard to give any meaning to subject behavior there.

**Table 4:** Endogenous Close. Output Choice. Forward vs. Spot Markets.

	Observed Forward	Observed Spot	Observed total
Duopoly	97.48	0.16	97.64***
Quadropoly	116.24	0.22	116.46***

Wilcoxon test: *** significantly different from perfect competition (100) at 1% level.

Although the total quantity does not change much along the experiment (there are no significant differences), we nevertheless observe three related trends as the experiment progresses. One is that the average number of forward market openings decreases from around 14 in the first rounds to less half that number towards the end (see Fig 2). That this has no major consequences on total quantities is due to the fact that sales after the fourth forward market are very small in any case. The second is that, as the experiment progresses, sales accumulate more in the two earliest openings of the forward market, from 65.08% in the first 10 periods to 86.87% in the last ten. The third is that the mark up goes down from 14.3, 9.9, 6.1, and 4.1 in the first four openings of the forward markets in the first 10 rounds of the experiment to 8.7, 4.3, 2.3, and 1.6 in the last ten. All these changes are statistically significant at 1%.

The evolution of prices is of particular import to our hypothesis. The hypothesis of collusive behavior implies no use of forward market and Cournot behavior in the spot market. Any small quantity sold in a forward market would be traded at or near to, the Cournot price (as the price in forward markets are computed as the Cournot price in the residual demand). However, what we find is that large quantities are sold in the forward markets and, consequently, the observed experimental price approaches the competitive price. The collusive hypothesis is clearly rejected from our experimental results.

The alternative hypothesis that players behave competitively implies that the price in all forward markets must be competitive, and the corresponding mark up, zero. This is neither accepted nor rejected, as this price is not observed. However, even if the experimental price is set equal to the Cournot price in the residual demand, the fact that large quantities are sold in the early openings of forward markets makes for a smaller residual demand as markets open, with a price that indeed approaches the competitive price. This is even more so in the last rounds of the experiment. In other words, the price in the residual demand converges to the competitive price. In this sense the experimental behavior converges to the competitive hypothesis.

We can see this in Fig 2, where the horizontal axis represents the openings of the forward market (note that opening 37 is spot market), and in the vertical axis we have the mark-up. Notice that the average mark up is computed conditioned on the forward market being open. For instance, if two duopolies trade in forward periods 1, 2, 3 and 4, and another one in forward periods 1, 2 and 3 the average mark up for period 4 is computed taking the average of only the first two. This clearly over represents the mark up in openings that are not reached in most of the duopolies. One could make the mark up zero for those openings, but we do not know that this would be the mark up if the market opened (although we know that it must be very low, as the residual demand is also very low). In any case, it is only in few occasions that the forward market continues beyond the 7^th^ opening.

### 4.2 Endogenous close: Quadropoly

The average quantity produced in the forward stages is 116.24% of the competitive quantity, and the spot quantity is only 0.22 (Table 4). The total quantity is significantly different from the competitive quantity but not from the demand intercept. It seems that quadropoly competition is so strong that the players keep selling until there is no demand. Given that the marginal cost is positive, this would normally imply selling the last quantities at a loss, but the way the price is computed makes things different. We were interested in testing the Cournot equilibrium, which means that, in every forward period, the price is selected to be the Cournot price in the residual demand. When sales in forward markets exceed what can be supplied above cost, the program assumes that in the future firms will sell nothing and sets a price equal to marginal cost, as in perfect competition, because the perfect competition quantity has been reached. This means that, in the experiment, additional units above the competitive quantity are inconsequential for the sellers and have the same meaning as the competitive quantity.

**Fig 3:**
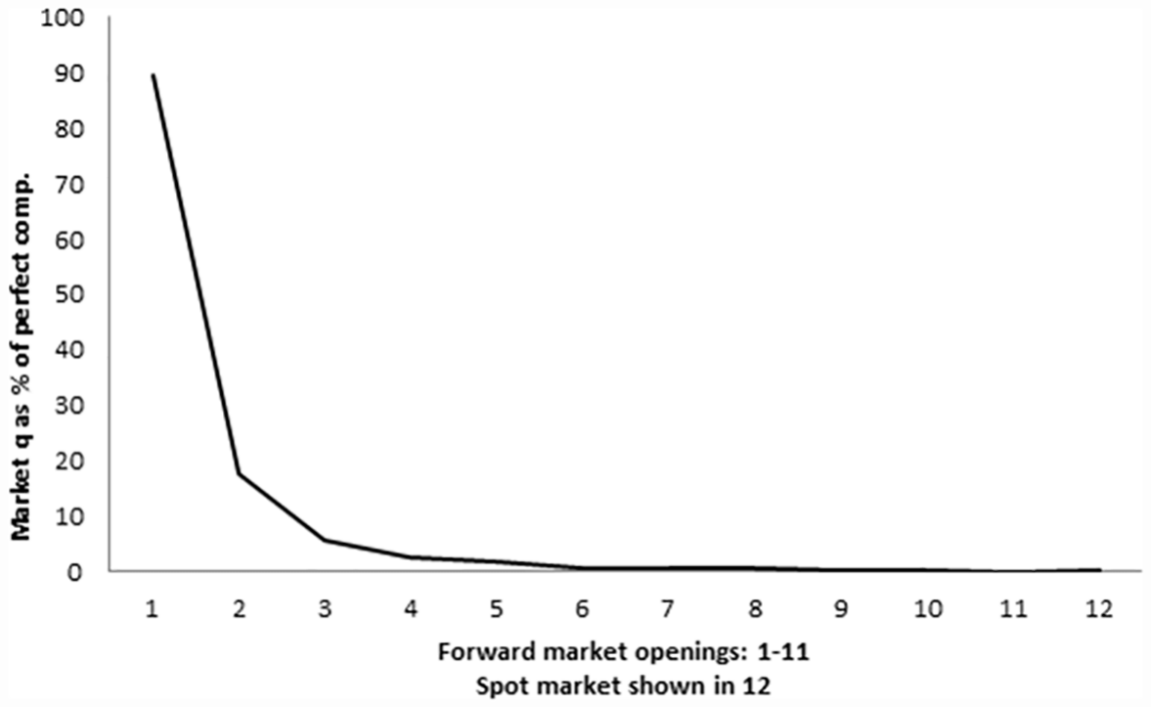
Use of forward and spot markets. Endogenous close, Quadropoly.

As under duopoly, sales take place almost entirely in the forward markets. The only difference is that the concentration in the first period is even greater (see Fig 3). No residual demand is left after the use of the forward markets. As a result there is nothing interesting to be seen in the spot market.

**Fig 4:**
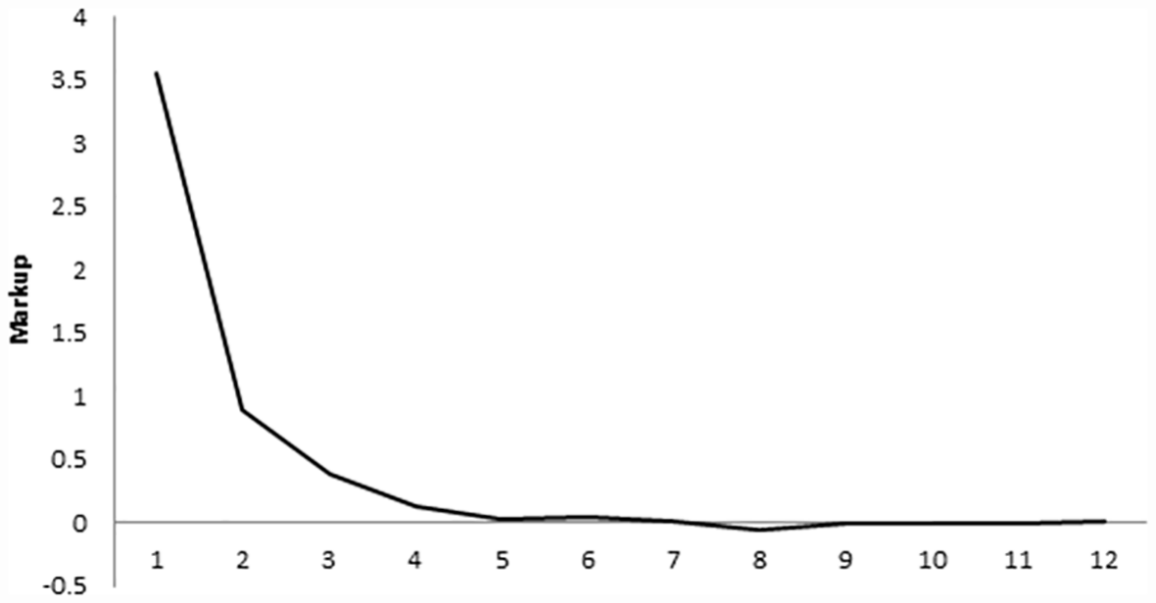
Markup per market opening. Endogenous close, Quadropoly.

The average mark up (price minus marginal cost) is 3.56 in the first opening of forward markets, 0.9 in the second, and 0.39 in the third. The mark ups in the other forward periods and in the spot market are near zero (Fig 4). By comparison, the mark up if the forward market were never used is 20 and the competitive mark up is zero. Although statistically different in the earliest periods, the realized prices come remarkably close to the competitive level even, which is remarkable as we compute them under the hypothesis that firms collude not to use the forward markets and play Cournot in the spot market. That is, although stronger, the same convergence to competitive behaviour as was observed under duopoly is observed here, with an average number of forward markets openings between 4 and 5.

Unlike in the duopoly case, subject behavior does not change much as the rounds unfold, neither in the number of forward openings nor in the average quantity chosen in the earlier periods of forward markets. The most plausible explanation for this difference is that competition is already very strong in the first periods and subjects do not find any mechanism to avoid it.

### 4.3 Exogenous close: Duopoly

In this section we provide a more detailed analysis and disaggregate the individual quantities among the two forward and the spot markets. In Table 5 we observe that the quantities chosen in the different markets and in the aggregate are remarkably in line with the theoretical predictions. Only the quantity chosen in the second period of opening of the forward market is significantly different from the theoretical prediction at the significance level of 5%. We use the Wilcoxon signed-rank test for the hypothesis of equal means.

**Table 5:** Exogenous Close, Duopoly: Use of forward and spot markets.

	Forward 1	Forward 2	Spot	Total
Theoretical quantity ex-ante	28.57	28.57	28.57	85.71
Theoretical quantity ex-post	28.57	26.1	30.54	-
**Average observed quantity**	34.75*	21.81**	**27.42**	**84**
	(20.87)	(7.42)	(14.58)	(11.04)

Wilcoxon Test: observation = theory ex-post

* Significant at 10% level.

** Significant at 5% level.

To understand better Table 5 notice that it compares observed quantities for the forward and spot markets against the theoretical *ex-post* quantity (given the observed quantity in the previous opening of the market). The theoretical *ex-post* prediction lists the subgame perfect equilibrium quantities in each stage. The theoretical predictions for the residual demand are computed as the subgame perfect equilibrium quantities in the subgame. For completeness we also show the theoretical *ex-ante* prediction (if players follow the equilibrium path).

Suppose that demand is *p = 100 –q*, costs are zero and 35 units are sold in the first opening of forward markets. Then the rest of the game is that of a one-period forward market with demand *p = 100–35 –q*. The theoretical prediction for the second period of forward markets in this sub-game is then computed. If the forward quantity in the second period is 20, the theoretical prediction in the spot market is the Cournot equilibrium in the duopoly game with demand *p = 100–35–20 –q*.

As a benchmark it is useful to see what would happen if firms behaved competitively or as a monopolist. If firms behaved as a monopolist in the residual demand of the spot market then the quantity would be (½)(100–34.75–21.81) = 21.72. However, if firms behaved competitively in the residual demand of the spot market, then the market quantity would be 43.44 (relative to the observed quantity of 27.42).

**Fig 5:**
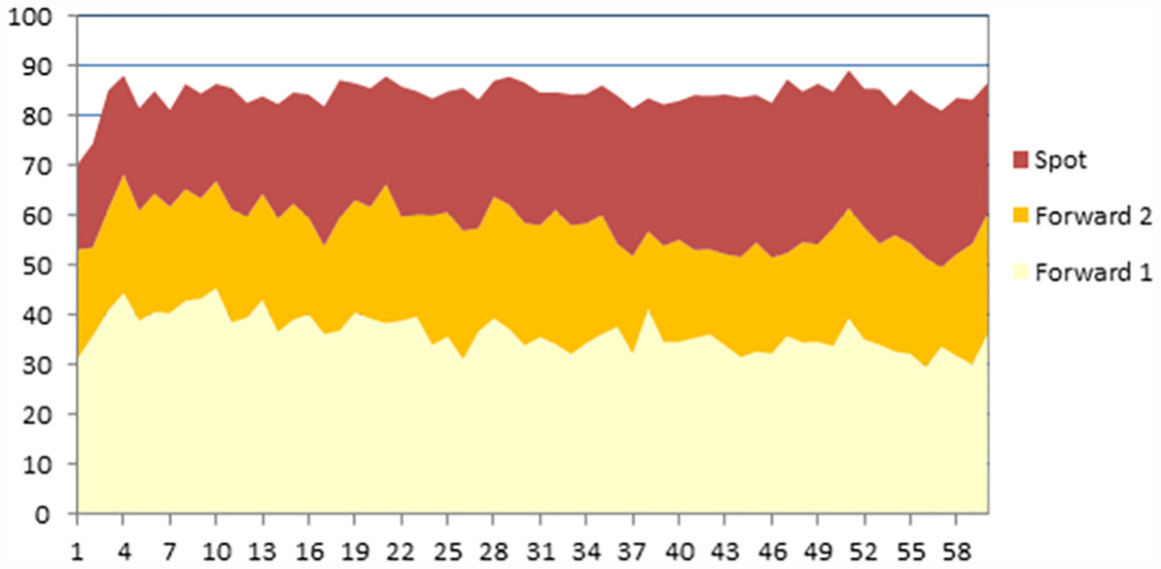
Use of Forward and Spot Markets. Exogenous Close, Duopoly.

Fig 5 below sheds some light on choices made by the subjects. We observe a slightly decreasing trend in the average quantities in forward markets as periods advance. To check this tendency we separate the data for the first and last 30 rounds (Table 6). First we notice that the change is never too big (the highest is 15%), and, second, that it is not significant for the spot market and total quantities. The change in forward markets is significant at 5%, but not at 1%.

**Table 6:** Exogenous Close, Duopoly. Use of forward and spot markets. First and Last 30 rounds.

		Forward 1	Forward 2	Spot	Total
	Theoretical quantity ex-ante	28.57	28.57	28.57	85.71
First 30	Theoretical quantity ex-post	28.57	24.97	26.57	-
	**Observed quantity (as proportion of ex-post theory)**	**37.57**	**22.57**	**24.18**	**84.32**
		**(131.52)**	**(90.41)**	**(94.66)**	**-**
Last 30	Theoretical quantity ex-post	28.57	27.23	31.35	-
	**Observed quantity (as proportion of ex-post theory)**	**31.93**	**21.05**	**30.67**	**83.64**
		**(111.77)**	**(77.3)**	**(98.77)**	**-**
	% Change in observed quantity as a proportion of theory ex-post	-15.01**	-14.5**	4.3*	-
	% Change in observation	-	-	-	-0.8

* Significant at 10% level.

** Significant at 5% level.

### 4.4 Exogenous Close: Quadropoly

Output is close to the theoretical prediction in both the forward and spot markets under a quadropoly (Table 7). This fact is even more remarkable than in the duopoly case because the theoretical equilibrium requires very different quantities in each of the markets. The exact theoretical prediction is only rejected for the spot market (at 5% significance level). The total theoretical quantity is very close to perfect competition, and observed quantity cannot be shown to be significantly different from either of them. Our results are along the lines of [2] where four or more agents behave more competitively than predicted by theory. Table 7 below shows this result.

**Table 7:** Use of forward and spot markets (Quadropoly).

	Forward 1	Forward 2	Spot	Total
Theoretical quantity ex-ante	67.92	22.64	7.55	98.11
Theoretical quantity ex-post	67.92	20.78	8.157	-
**Average observed quantity**	71.8**	**20.24**	9.33**	**101.37**
	**(33.88)**	**(8.17)**	**(1.14)**	**(27.33)**

** Significant at 5% level.

**Fig 6:**
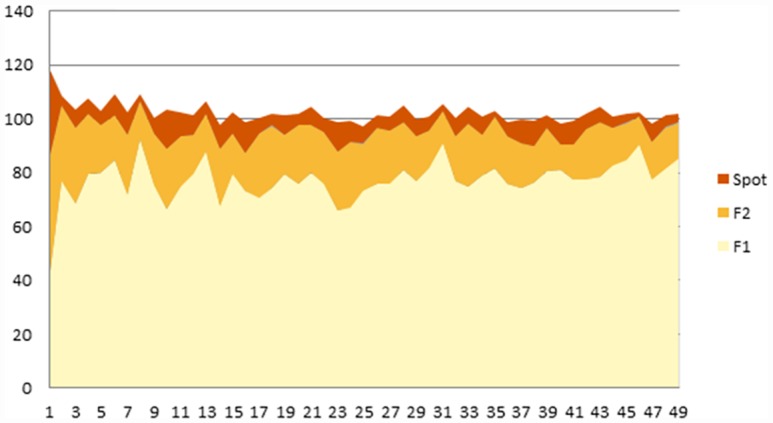
Use of Forward and Spot Markets. Exogenous Close, Quadropoly.

Fig 6 shows us the average output for quadropolies. We check whether there is a tendency in the data by comparing the first and second half of the periods. The hypothesis that there is no change in the behavior, when measured as the proportion with respect to theoretical quantity, is only clearly rejected for the spot market, and only rejected at 10% for the second forward market. The change in total quantity is small and also only significant at 10%. (Table 8.)

**Table 8:** Quadropoly Exogenous Close. Use of forward and spot-First and Last 21 rounds.

		Forward 1	Forward 2	Spot	Total
	Theoretical quantity ex-ante	67.92	22.64	7.55	98.11
First 21	Theoretical quantity ex-post	67.92	18.8	6.5	-
	**Observed quantity (as proportion of ex-post theory)**	**70.9**	**20.48**	**9.2**	**103.25**
		**(104.4)**	**(99.62)**	**(133.11)**	**-**
Last 21	Theoretical quantity ex-post (given observed quantity)	67.92	15.87	5.53	-
	**Observed quantity (as proportion of ex-post theory)**	**72.65**	**18.05**	**8.18**	**99.54**
		**(106.96)**	**(93.49)**	**(109.9)**	**-**
	% Change of observation	-	-	-	-3.59
	% Change of observation as a proportion of theory ex-post	2.46	-5.99*	-7.71***	-

* Significant at 10%.

*** Significant at 1%.

### 4.5 Increasing the number of firms vs. opening forward markets

In another paper [22] we report some experiments regarding Cournot oligopolies performed at the same universities. Below we compare some results from this paper with the exogenous and endogenous close forward markets experiments. We compare the effect of introducing more firms in the market, *i*.*e*., 2 vs. 4, and the addition of forward markets. Table 9 summarizes average sales by duopolies and quadropolies (with no forward markets) against the exogenous close and the endogenous close forward markets.

**Table 9:** Number of firms vs. Forward markets.

	Cournot	Exogenous Close with two forward markets	Endogenous Close
Duopoly	70	85.56	97.64
Quadropoly	84.58	102.4	116.46

This comparison shows that the experimental effect of introducing two periods of forward markets in a duopoly is similar to the effect of increasing the number of firms to four. [2] showed that when the number of futures markets is one, the effect of increasing the number of firms from two to four is higher than the effect of introducing one period of forward markets.

One may be tempted to assert that the introduction of forward markets with the endogenous close rule imposed in the experiment may be one of the keys to competition (Table 9). It is, however, premature to conclude this, as further empirical and experimental evidence is needed. For instance, in our setup, the forward price is set automatically by the program, whereas in a more complete experiment it should be determined endogenously with active buyers. Clearly, this may change subjects’ behavior. For instance, if the price trend along the forward markets is as observed in our experiments, buyers might realize that if forward markets are used the price will tend to zero, and then they will not be willing to buy in the early periods and will wait until later ones. Once this is learned, sellers will not sell in the forward market. This may enable the players to coordinate on the Cournot outcome. Of course, this is only a hypothesis, but one that suggests that more experimental designs should be tested. As with any hypothesis that deals with learning, experiments that used experienced subjects should also be conducted.

## 5. Conclusion

There is some controversy about the impact of the introduction of forward markets on market competitiveness. They are widely used and little understood. Depending upon the model, theory provides results suited to all tastes. The introduction of forward markets can have pro- and anti-competitive effects. The experimental literature is still scant, but points towards the competitiveness of forward markets. For example, [2] provide some support for theory for a duopoly and stronger support for a quadropoly. [3] meanwhile, show that the introduction of forward markets can result in competitive outcomes. [4] also show that the effect of an extra forward market is stronger than adding one more competitor.

We design our experiments to test the effect of multiple forward market openings on market competitiveness. We first test an endogenous close model that can potentially have indefinitely many forward markets. Our model is an extension of [1]. We find that the endogenous close experiments are extremely competitive and the price in the residual demand converges towards the competitive price both for duopolies and quadropolies. The hypothesis that firms would avoid the use of forward markets to play Cournot in the spot market is rejected in these experiments.

We also test an exogenous close model with two forward markets. Average output in our experiments is remarkably close to the theoretical prediction in both the final total quantity and the use of forward markets. Output produced by our subjects does capture the prisoners’ dilemma nature of the strategic motive to sell forward. Further, behavior in the forward and spot phase in our experiments corresponds more to the theoretical prediction. This is especially the case under duopolies.

Our experimental results suggest a number of further research avenues. First, forward markets are complicated and it seems important to assure that the subjects understand the structure. For example, it seems that subjects find it very hard to sell zero amounts in the forward stage. In view of this the effect of subject experience seems important. There are a couple of works [4], [22] on forward market experiments with experienced subjects. Their results are contradictory, as the first report that experienced subjects are less competitive, while the second shows the opposite. Second, the experimental design relies on the imputation of a price to quantities sold in the forward market, which is done by using the theoretical predictions. A more complete design that allows the endogenous determination of this price will solve this problem [3]. Third, the introduction of arbitrageurs that can freely buy and sell would add a more realistic feature to the experiment. We are working on these issues currently.

## Supporting Information

S1 File Instructions. (PDF)

S2 File Theory. (PDF)
