# Supplementary material for: Multiple Openings and Competitiveness of Forward Markets: Experimental Evidence
Source: PLoS One. 2016 Jul 21;11(7):e0158098. doi: 10.1371/journal.pone.0158098 (PMC4956257; doi:10.1371/journal.pone.0158098)
Supplement: S1 File — (PDF) [file pone.0158098.s001.pdf]

S1 File. Instructions.

Introduction: This is a study of decision-making. Funding for this project has been provided by public funding agencies. If you follow these instructions, and make decisions carefully, you might earn a considerable amount of money. You will be paid **IN CASH** at the end of today's session.

Important: At any stage you can raise your hand to ask any question relating to the experiment.

Overview: In today's session each of you is a quantity-setting seller. There are TWO sellers in each market. The experiment is made up of several **weeks.** Each **week** is made up of three **trading days**. You will be randomly and anonymously matched against other opponents.

Trading in each week proceeds as follows:

Each week is made up of **three days**. Note that, the total of the offers made in all the days constitute a **commitment** to sell a good and are final. **In each day you make profits for the quantities sold in that day only**.

First Day:

In the first day you will have 30 seconds to make quantity offers. Note that, once confirmed all **offers to sell** are FINAL and cannot be changed. At the end of the day you will be able to see the quantities offered by the other seller, the price, and your profits **for the day**.

Second Day:

You may choose, or not, to **increase** upon the offer you made in the first day. You will have 30 seconds to make quantity offers. Any change can only be an increase over the total quantity offered in day-1. At the end of the day you will be able to see the quantities offered by the other seller, the price, and your profits **for the day (only for the additional quantity sold in this day)**.

Final Day:

This is the final day of the week. You may choose, or not, to increase the offer you made in the first two days. Any change can only be an increase over the total quantity offered in days -1 and -2. At the end of the day you will be able to see the quantities offered by the other seller, the price, and your profits **for the day (only for the additional quantity sold in this day)**.

You can offer to sell quantity in all, or any, of the days. The **price** received by sellers is the **same for everyone.**

- The market price in Day 1 is determined by the sum total of quantity offered by ALL sellers during that day and a **computer estimate of the quantity that will be sold on Day 2 and the Final Day**.
- The market price in Day 2 is determined by the sum total of quantity offered by ALL sellers during Days 1 and 2 **and a computer estimate of the quantities for the Final Day**.

Example 1 below explains how the price is determined in **the Final day**.

Example 1: Let the market demand be P=10-TQ (P = market price, TQ = total quantity offered by all sellers). Suppose you offered to sell **ZERO units** on **day-1, ONE additional unit** on **day-2, and ONE on Final day**. The sum total of the units offered by you then is 2 (=1+1).

Let us also suppose that the number of units offered by the other seller on **day 1** is 1**,** 1 on **day 2, and ZERO on the Final Day**. The total quantity (TQ) offered by all sellers across the week then is (3+2=) 5. This implies that the market price for the Final Day is P = 10-TQ = 10-5 = **5**.

Note that the price declines as the total quantity offered (TQ) increases. For all TQ greater than, or equal to, 10 the market price (P=10-TQ=10-10=0) is zero. **Further note that, the market price can never be negative.**

Example 2 below explains the relationship between the total quantity offered (TQ) and the market price in the Final Day (P).

Example 2: Notice that the market price (P=10-TQ) decreases as the total quantity (TQ) sold in the market increases. The table below gives some possible prices for the Final Day for different total quantities (TQ):

Market demand: P=10-TQ	
QUANTITY (TQ)	PRICE (P)
1	P = 10-1 = **9**
2	P = 10-2 = **8**
4	P = 10-4 = **6**
6	P = 10-6 = **4**
7	P = 10-7 = **3**
8	P = 10-8 = **2**
9	P = 10-9 = **1**
10	P = 10-10 = **0**

Procedures for trading are explained in more detail below.

1. Sellers earn profits by selling units. The profit for any unit sold is the selling price minus the cost of the unit. The selling price will be the same for all units, as will be unit costs. Thus a seller's total profit is;

Profits in the Final Day = (Selling Price – Unit Cost) × Number of units sold in the Final Day

2. Buyers. The buyers are automated. The price is determined according to the demand in **Example-1**. Given total quantity (TQ), the market price P=10-TQ. In our example TQ=5, this implies that P = 10-TQ = 10-5 = 5.

Note that the same demand will not be used in the experiment.

In Days 1 and 2, the price is computed by the computer. As explained before the **computer estimates the quantity that will be sold on Day 2 and the Final Day**.

Before you confirm your quantity for the day, you can practice with different quantities for yourself and for the other seller (to have an estimate of the effects on your profits of the total quantity offered that day).

There are several important things to understand.

- The higher (lower) is the total quantity (TQ), the lower (higher) is the price (P)

(see **TABLE** in **Example 2** above).

- Your sales are affected by the quantities chosen by the other seller. The higher (lower) is the other seller's quantity lower (higher) is the sales price. The same will be true if you increase your quantity and the other seller does not.

- **A higher quantity today may increase your profits today but may decrease profits later on in the week.**

<u>The trading week:</u>

Each seller can offer to sell some quantity (or none) in each day of the week. While choosing the quantity you should keep in mind that,

(i) you earn profits by selling units at a price above Unit Cost and
(ii) the higher is total quantity, the lower is the sales price (see **table** above).
(iii) you earn zero if you sell nothing.

<u>How to read the screen and submit your offer?</u>

On the right side of the screen, there is a **history table**. A record of all the plays is displayed in the table.

On the left side of the screen, there is a **graphical display** section.

You can try different possible combinations of your offer, the sum of all the other sellers offers and observe your potential profit **on the right side of the display section**.

After you have decided your offer for that day, click the CONFIRM button. NOTE that whenever you click the CONFIRM button, you are confirming **your offer only**. The actual number of units offered by other sellers may be different from yours. Also, NOTE that you **must** click the CONFIRM button in order to submit your offer.

The left side of the graphic display section shows your quantity, the sum of other sellers' quantity and the profit given the price on a particular day.

4) Overview:

a) Today's experiment will consist of a number of **weeks**. A trading week is made up of **three days**. The final trading week will not be disclosed in advance.

b) Each of you can choose to offer a quantity for sale in any trading day. You will be randomly and anonymously matched against other opponents.

c) In today's experiment each one of you will have a Unit Cost of $X in each period. Each participant has identical Unit Costs, and Unit Costs are the same in all trading weeks. You are also informed about the other seller's Unit Costs in a history table on the Right Side of the screen.

d) You will be paid $X U.S. for every Y "experimental dollars" you earn in the market. Thus, for example, every Y experimental dollars equals $U.S. Your total earnings for today's session will be the sum of your earnings in the experiment, plus your appearance fee.

e) Some participants may make their quantity decisions earlier than others. If you make your decision before other sellers, please wait quietly while others finish. The monitor will make sure that there are no unnecessary delays.

f) Please note that, talking with, or looking at, other participants is not allowed. The market will be closed and all participants will be dismissed without further payment if any participant communicates in any way other than the manner described in these instructions.

g) At the end of the experiment you will be called out and your earning will be paid to you in cash.

You will now practice before you start the experiment. Please free feel to continue the practice until you are ready for the experiment. Please click on “Ready to Practice” if you fully understand the instruction.
