# Supplementary material for: Multiple Openings and Competitiveness of Forward Markets: Experimental Evidence
Source: PLoS One. 2016 Jul 21;11(7):e0158098. doi: 10.1371/journal.pone.0158098 (PMC4956257; doi:10.1371/journal.pone.0158098)
Supplement: S2 File — (PDF) [file pone.0158098.s002.pdf]

S2 File. Theory.

Theory does not seem to be in agreement regarding multiple market openings and whether they result in competitive outcomes. Given this we focus on [1] – [3]. [1] shows that, if firms can sell in a forward market previous to the spot market, the strategic interactions result in a more competitive outcome. In another paper, [2] show that this pro-competitive effect increases as the forward markets open more often. Further, as the number of forward market openings goes to infinite, the quantity tends to the competitive outcome.

On the other hand, [3] argues that if the forward market has infinitely many moments in which trade is allowed, any price between Cournot and perfect competition can be sustained in equilibrium. As in many other instances, the limit of the equilibria in finite games may not exhaust all the equilibria in the infinite game. In fact, something similar to a Folk Theorem is obtained if the infinite case is analyzed directly. In this case, any total quantity (and their corresponding market prices) between competitive and Cournot can be observed in equilibrium. Note that the Cournot result can be supported in equilibrium by the following strategy. Firms sell nothing in the forward markets and play standard Cournot in the spot market. If a firm deviates and sells forward at some point, the other firms also sell in the next period. When one firm sells forward, it makes some extra profits with respect to the equilibrium behavior. However, when the other firms also sell in the next period to punish the deviation, its profits are reduced. The punishment phase is calibrated so the deviator makes a net loss. [3] shows that similar strategies can actually support any outcome between the competitive and the Cournot quantities. However, the Cournot outcome is the only one that satisfies some equilibrium refinements like renegotiation-proofness or Pareto perfection.

Notice that after firms sell in the forward market, each of the subgames is a reduced version of the original game (with a smaller residual demand, depending on how much was sold in the previous markets). This makes the model different from a repeated game because, in the repeated game, the demand remains the same in each period. There is, however, a similar result once it is established that there is still room for credible punishments in spite of the smaller demand and of the smaller impact of the punishment.

Next, we outline two versions of forward markets. For didactical purposes we present first the model in [2], where the number of forward markets is exogenously determined.

2.2 Allaz and Vila (1993)[2]

Suppose there are *n* firms in an oligopolistic market that compete in quantity and face a linear demand $p = A - q$ with zero costs. If, previous to this spot market, firms can sell forward, in the Nash-Cournot equilibrium, Firm *i* will sell $s_i = \frac{A-F}{n+1}$ in this spot market, where *F* is the total of quantities sold in the forward market. The equilibrium price will be $p_s = \frac{A-F}{n+1}$.

If there are 2 periods of forward markets, in period $t = 2$ Firm *i* will solve the problem

$$\max_{f_i^2}(f_i^2 + s_i)p_s,$$

where $s_i = \frac{A-F}{n+1}$, and $p_s = \frac{A-F}{n+1}$.

Taking into account that now $F = \sum_{j=1}^{n} f_j^1 + \sum_{j=1}^{n} f_j^2$, with f_j^t as the quantity sold by Firm *j* in the forward market at time *t*.

We assume a no-arbitrage condition in solving this problem. This implies that forward and spot prices are equal. For example, [1] shows that the introduction of arbitrageurs that buy in the forward markets to sell in the spot implies that there is no arbitrage in equilibrium. Substituting the arbitrageurs with the no-arbitrage condition gives the same results and simplifies the model. The solution of the problem for each firm gives the solution

$$f_i^2 = \frac{n-1}{n^2+1}(A - F^1), \text{ and } s_i = p_s = \frac{1}{n^2+1}(A - F^1),$$

where $F^1 = \sum_{j=1}^{n} f_j^1$.

Now, in period 1 of the forward market, Firm *i* solves

$$max_{f_i^1}(f_i^1 + f_i^2 + s_i)p_s,$$

where $f_i^2 = \frac{n-1}{n^2+1}(A - F^1)$, and $s_i = p_s = \frac{1}{n^2+1}(A - F^1)$.

The solution of this problem for all firms gives

$$f_i^1 = \frac{(n-1)^2 A}{n^3-n^2+n+1}.$$

The rest of the variables are found substituting this value in their corresponding expressions. When firms face identical, constant marginal costs *c*, $A - c$ replaces A in

all of the above expressions, and the price will be given by the expression, $p_s + c = \frac{1}{n^2+1}(A - c - F^1)$.

2.3 Extensions of Allaz and Vila (1993)[2]

Allaz and Vila examine a model with finitely many periods of forward markets and find that, as the total number of periods increases, the total sold quantity also increases. Further, as the number of periods of forward markets goes to infinite, the limit of the quantity is the competitive outcome. For the particular case of two firms, the case of *T* periods in which the forward market is open, gives

$$p = s_i = f_j^t = \frac{A}{3+2T}, \text{ and} = 2\frac{(1+T)A}{3+2T}.$$

It can easily be checked that, as *T* increases, the price *p* goes to zero, and total quantity *q* converges to *A*, the competitive outcome.

2.1 The endogenous close rule.

We implement an adaptation of the specification in [3]. First, note that we cannot have infinitely many forward markets periods in our experimental setup. Due to this we replace the condition in the model of [3] with the following adaptation: forward markets are open at period 1, and remain open at period t as long as total positions at period $t-1$ were positive. Otherwise the forward market is closed and the game goes to the spot market. Note that, this condition does not change the predictions of [3]. The second adaptation has to do with the integer problem (as subjects cannot enter quantities with decimals). This changes the model, but only in the sense that the results in [3] are approximations of the results in the model with the integer restriction. For the sake of completeness, here we show that under the endogenous close rule both Cournot and competitive prices can be sustained in a subgame perfect equilibrium in the adapted model in the same fashion as they are sustained in the original model.

Consider the case of one forward market before the spot market and demand given by $p = n - q$ where n is a natural number. The forward market opens at discrete times, and each time firms can choose to sell any amount in the market (after observing the previous positions). The forward market opens at time t if some quantity was sold at $t-1$, otherwise it closes and the game goes to the spot market. Firms strategy choice is $f_i^t, s_i \in \{0,1,\dots,n\}$, where f_i^t is the forward quantity sold by Firm i in forward period *t* and s_i is the spot quantity sold by Firm *i*.

The Competitive equilibrium:

In the forward market firms play the following way:

(i) Firm i chooses $f_i^t = \frac{n-\sum_{t,k} f_k^{t-1}}{2}$ if $n - \sum_{k,t} f_k^{t-1} \geq 2$ and even,

(ii) Firm i chooses $f_i^t = \frac{n-\sum_{t,k} f_k^{t-1}+1}{2}$, and Firm j chooses $f_j^t = \frac{n-\sum_{t,k} f_k^{t-1}-1}{2}$, if $n - \sum_{k,t} f_k^{t-1} \geq 2$ and odd,

(iii) Firm i chooses $f_i^t = 1$, and Firm j chooses $f_j^t = 0$ if $n - \sum_{k,t} f_k^{t-1} = 1$,

(iv) both firms choose $f_i^t = 0$ if $n - \sum_{k,t} f_k^{t-1} = 0$.

In the spot market firms play Cournot in the residual demand which, in the equilibrium path, is $s_1 = s_2 = 0$. In all cases, forward and spot market prices are $p_f^t = p_s = p = 0$, and profits are also zero. Given the strategy chosen by the opponent, to change the forward quantities by a given player does not change the price and no positive profits can be expected in any market.

The Cournot equilibrium:

In the forward market firms play:

(i) $f_i^1 = 0$,

(ii) at $t > 1$ play as in the competitive equilibrium,

(iii) in the spot market play Cournot in the residual demand which, in the equilibrium path, firms play one of the integers among the closest to $\frac{n}{3}$.

Clearly, by following the strategy, firms get a non negative profit (positive if $n \geq 3$). Any deviation in the forward market results in zero profits. In the spot market firms play according to equilibrium. The case for the quadropoly is similar.

Supporting Information References

1. Allaz B (1992) Oligopoly, uncertainty and strategic forward transactions. Int J Ind Organ 10: 297-308.

2. Allaz B, Vila JL (1993) Cournot competition, forward markets and efficiency. J Econ Theory 59: 1-16.

3. Ferreira JL (2003) Strategic interaction between futures and spot markets. J Econ Theory 108: 141-151.
